# Croup

**Published:** 1901-03

**Authors:** 


### Croup.

℞ Carbonate ammon ................gr. ii.
Syr. of senega .....................m. x.
Mucilage of acacia................dr. ii.

M. Sig.: To be given every two hours, unless patient vomits; if vomiting occurs, diminish the dose.

Also in steam atomizer:

℞ Sodium carbonate, dr. i—dr. iiss.
Aq., q. s. ad. oz. iv.

M. Sig.: Every half hour or oftener.—*Wharton, Ex.*

